# Consumption of Distinct Dietary Lipids during Early Pregnancy Differentially Modulates the Expression of microRNAs in Mothers and Offspring

**DOI:** 10.1371/journal.pone.0117858

**Published:** 2015-02-11

**Authors:** Patricia Casas-Agustench, Flavia S. Fernandes, Maria G. Tavares do Carmo, Francesco Visioli, Emilio Herrera, Alberto Dávalos

**Affiliations:** 1 Laboratory of Disorders of Lipid Metabolism and Molecular Nutrition, Madrid Institute for Advanced Studies (IMDEA)-Food, CEI UAM+CSIC, Madrid, Spain; 2 Instituto de Nutrição Josué de Castro, Universidade Federal do Rio de Janeiro, Rio de Janeiro, Brazil; 3 Department of Biochemistry and Chemistry, Faculties of Pharmacy and Medicine, Universidad San Pablo CEU, Madrid, Spain; University of Southampton, UNITED KINGDOM

## Abstract

Diet during pregnancy and lactation influences the offspring’s health in the long-term. Indeed, human epidemiological studies and animal experiments suggest that different type of fatty acids consumption during pregnancy affect offspring development and susceptibility to metabolic disorders. Epigenetic changes are thought to be elicited by dietary factors during critical timing of development. microRNAs (miRNAs) are versatile regulators of gene expression. Thus, we aimed to determine the influence of different fatty acids on miRNA expression in offspring when given during early pregnancy. We fed pregnant either soybean (SO), olive (OO), fish (FO), linseed (LO), or palm-oil (PO) diets from conception to day 12 of gestation; and standard diet thereafter. miRNA expression was assessed in liver an adipose tissue of pregnant rats and their virgin counterparts. While liver concentrations of fatty acids in pregnant or virgin rats replicated those of the diets consumed during early pregnancy, their pups’ liver tissue marginally reflected those of the respective experimental feeds. By contrast, the liver fatty acid profile of adult offsprings was similar, regardless of the diet fed during gestation. Different parental miRNAs were modulated by the different type of fatty acid: in adult offspring, miR-215, miR-10b, miR-26, miR-377-3p, miR-21, and miR-192 among others, were differentially modulated by the different fatty acids fed during early pregnancy. Overall, our results show that maternal consumption of different types of fatty acids during early pregnancy influences miRNA expression in both maternal and offspring tissues, which may epigenetically explain the long-term phenotypic changes of the offspring.

## Introduction

Human epidemiological studies as well as a variety of animal ones revealed that prenatal and early postnatal nutritional statuses may influence adult susceptibility to impaired glucose tolerance, cardiovascular disease, and obesity [1–4]. However, little is known about the mechanism(s) underlying these phenomena [4]. While increasing evidence suggests that maternal fatty acid status during pregnancy and lactation greatly influences newborn and infant health [5,6], very few studies have paid attention to the long-term consequences of changing the maternal dietary fatty acid composition [5,7,8]. Different dietary fatty acids modulate different biologically-relevant pathways [9,10]. As an example, a recent study in rats demonstrated that the intake of high amounts of n-3 fatty acids compared to other types of fatty acids during early pregnancy reduces fat accretion and age-related decline in insulin sensitivity in male offspring [5]. However, the precise nature of these effects remains elusive.

In addition to diet, epigenetic modifications may influence gene expression and modulate the phenotype of the organism much later in life, via exposure to an altered intra-uterine milieu or metabolic perturbation [11–13]. MicroRNAs (miRNAs) are small endogenous non-coding RNAs that regulate several cellular and biologic processes by regulating gene expression [14]. By targeting complex biological pathway, miRNAs ‘fine-tune’ gene expression under physiologic conditions, but it is under conditions of stress when their function becomes especially pronounced, underscoring their role in health and disease [15].

Fatty acid supplementation during development could affect epigenetic gene regulation by inducing (or repressing) transcription of specific genes during critical ontogenic periods, during which tissue-specific marks may be initiated [16]. Therefore, studies on the potential epigenetic mechanism involved in these findings are warranted. Although the evidence that fatty acids affect epigenetic gene regulation is limited, several findings indicate that the quantity and quality of fatty acids consumed by mothers during pregnancy and lactation can induce permanent changes in gene expression and metabolism in their offspring [17,18]. There is in vitro evidence that certain fatty acids could modulate the expression of miRNA in different cell types [19–22]. However, in vivo evidence of any one of these effects is limited [7,23]. It was recently shown that maternal consumption of a high-fat diet affects early lipid metabolism of offspring by modulating the expression of hepatic β-oxidation-related genes and that miRNAs can contribute to metabolic disturbances in adult life [7]. Whether this effect is influenced by the type of fatty acids used in the diet was not evaluated, but these results suggest that a diet containing high proportions of certain fatty acids could modulate the expression of specific miRNAs in the offspring, which may in turn lead to metabolic disorders.

The present study was designed to investigate whether consumption of different types of fatty acids during the first 12 days of pregnancy in rats influence the expression of miRNAs in parental tissues and whether these precocious exposures influence miRNAs expression in offspring. Depending on the tissue evaluated, different parental miRNAs were found to be modulated by different type of fatty acids. Even after 12 month after switch to a normal chow diet, offspring adult liver exhibited a miRNA expression influence by the early exposure.

## Materials and Methods

### Animals, diets, and experimental design

The experimental protocol was approved by the Animal Research Committee of the University of San Pablo-CEU (protocol number PDC 054). This research conforms to the institutional and national guide for the care and use of laboratory animals. Female Sprague-Dawley rats were obtained from the animal facility of the University San Pablo-CEU, Madrid. Rats were initially fed a standard non-purified diet (B&K Universal, Barcelona, Spain) and were housed under controlled light and temperature conditions (12-hour light/dark cycle; 22 ± 1°C). Rats were mated when they were three months old. On the day that semen appeared in vaginal smears (day 0 of pregnancy), rats were randomized and assigned to one of five dietary treatments, which differed from each other only in their non-vitamin lipid components (S1 Table). The soybean oil (SO) diet contained 9% soybean oil; the olive oil (OO) diet contained 9% olive oil; the fish oil (FO) diet contained 8% fish oil plus 1% sunflower oil; the linseed oil (LO) diet contained 8% linseed oil plus 1% sunflower oil; and the palm oil (PO) diet contained 8% palm oil plus 1% soybean oil as the only non-vitamin lipophilic component. Sunflower or soybean oil was added to some diets to meet the minimum requirement for essential fatty acids. The experimental diets were isoenergetic (4.1 kcal/g) and their composition and proportion of fatty acid profiles are shown in S1 Table. The diets were prepared at the beginning of the experiment and were kept at -20°C fractioned in daily portions. Every 24 h, fresh diet was provided and daily food intake was estimated periodically. After 12 days on an experimental diet (day 12 of pregnancy), 6–8 rats from each group were sacrificed and the remaining pregnant rats were given standard pellet diet (B&K Universal, Barcelona, Spain) until the end of the study. During lactation, pups were maintained with their mothers until weaning and all litters were culled to 8 pups per dam within 24 h of delivery, by selecting the excess number of pups at random and removing them. Rats always had free access to the assigned diet and tap water. After being weaned (postnatal day 21), pups were housed in collective cages (3–4 per cage) and were given the standard pellet diet until the end of the experiment. Age-matched virgin female rats, subjected to the same dietary regimens for the first 12 days of experiment as the pregnant rats, were studied in parallel. Rats were sacrificed by decapitation and after their exsanguinations liver and lumbar adipose tissue were dissected, placed in liquid nitrogen, and, after weighing, kept at -80°C. Offspring at 1 day of age and male adults of 12 months were sacrificed and their liver was collected as indicated above. Liver and adipose tissues were used for miRNA analysis.

### Processing of samples

**Determination of composition of fatty acids**. Nonadecaenoic acid (19:1; Sigma Chemical Co., St. Louis, MO, USA) was added as the internal standard to fresh aliquots of each diet and frozen livers and lumbar (retroperitoneal) adipose tissues were used for lipid extraction and purification [24]. The final lipid extract was evaporated to dryness under vacuum and the residue suspended in methanol/toluene (4:1 by volume) and subjected to methanolysis in the presence of acetyl chloride at 80°C for 2.5 h, as previously described [25]. Fatty acid methyl esters were separated and quantified in a Perkin-Elmer gas chromatograph (Autosystem) with a flame ionization detector and a 30 m BPX capillary column (internal diameter 0.25 mm). Nitrogen was used as the carrier gas and fatty acid methyl esters were identified by comparison with authentic standards (Sigma Chemical Co., St. Louis, MO, USA). Coefficient of variation values expressed as percentages ranged between 0.0 and 6.0, giving a mean ± ES value of 2.28±0.26% for an arbitrary set of fatty acid analyses.

### miRNA analysis

Total RNA, including miRNAs, was extracted using the miRNeasy minikit (Qiagen, Valencia, CA, USA), following the manufacturer’s instruction. Whole tissues were homogenized before RNA extraction. RNA was quantified with a NanoDrop ND-2000 UV-Vis spectrometer (Thermo Scientific, Wilmington, DE, USA). For rat whole genome miRNA analyses, complementary DNA (cDNA) was synthesized using the Universal cDNA synthesis kit II (Exiqon, Vedbaek, Denmark). miRNAs were analyzed by real time qPCR (qRT-PCR) using the rat miRNome miRNA ready-to-use PCR panels and ExiLENT Sybr green master mix (Exiqon, Vedbaek, Denmark), following the manufacturer’s instructions. miRNome analysis was assessed in 4 samples per group. For individual miRNA analysis, cDNA was synthesized using miScript® II Reverse Transcription kit (Qiagen, Germantown, MD) according to the manufacturer’s guidelines. Individual miRNAs were quantified by qRT-PCR using ABI Prism 7900 Sequence Detection System (Life Technologies, USA). RNU43, RNU6 and/or RNU5G were used as housekeeping genes and the analysis of relative gene expression was analyzed using the 2(-Delta Delta C(T)) method [26] and using the Exiqon GenEx qPCR analysis software (Exiqon, Vedbaek, Denmark).

### Gene expression analysis

Expression (mRNA) of insulin signaling related genes were analyzed by qRT-PCR using the rat insulin signaling pathway PCR array (SABiosiences-Qiagen, Hilden, Germany), following the manufacturer’s instructions. 5 animals were analyzed per group. Actin was used as housekeeping gene and relative gene expression was analyzed as described above.

### Statistical analysis

All data are expressed as means ±SE. Data were tested for normal distribution with the *Kolmogorov-Smirnov* test and statistical differences between dietary groups were measured by one-way ANOVA with Newman-Keuls pos hoc or Kruskal-Wallis, followed by Dunnet correction for multiple comparisons. Differences between two variables were measured with t- or Mann-Whitney tests, according to the normality of data’s distribution. We used the SPSS statistical software v.17. GenEX v.2.6.4. software (MultiD Analyses AB, Göteborg, Sweden) was used to manage the qRT-PCR data and GraphPad Prism 5.01 software (GraphPad Software Inc., San Diego, CA, USA).

## Results

### Nutritional parameters, body and tissue weights

Body weight and food intake were higher in pregnant rats than in virgin rats, but no difference was found between the groups of pregnant or of virgin rats receiving the different dietary treatments as previously reported [27]. Body weight of offspring at birth was not different between dietary groups [25]. However, as previously reported [5], the weight of lumbar adipose tissue in males at 12 month of age was lower in the FO group that in the others. This difference disappeared when corrected for body weight, although adipose tissue mass per 100 g of body weight was significantly (p < 0.01) lower in the FO group compared with the SO, OO, or PO groups, yet not with the LO group. Liver weight was lower in the FO group than in the PO one, but this difference also disappeared when corrected by body weight [5].

### Hepatic fatty acid concentrations

Concentrations of fatty acids in liver tissue of virgin and pregnant rats at day 12 on their respective diet, i.e. on the 12^th^ day of pregnancy are shown in Table 1. As expected, the fatty acids composition of liver tissues of rats fed the experimental diets during the first 12 days of pregnancy closely reflected the fatty acids composition of the diets eaten by each group (S1 Table). The same applied to lumbar adipose tissue, as we previously described [27]. Virgin rats also exhibited a similar pattern of fatty acid composition in the liver (Table 1). Therefore, there were no major differences among fatty acid profiles between pregnant vs. virgin within the same treatment. As expected, PO exhibited the highest amounts of palmitic and saturated fatty acids and OO the highest amounts of oleic and monounsaturated fatty acids. Concerning arachidonic acid, we observed decreased concentrations in FO and LO, possibly due to the competitive inhibition of Δ6-desaturase [28] as a consequence of the increased concentration of both DHA and EPA in these groups. In fact, the FO and LO groups had the highest hepatic concentrations of both EPA and DHA, as compared with the other groups, although alpha-linolenic acid was found in significantly higher amounts only in LO. Hepatic fatty acid concentrations of one day-old pups and one year-old offspring are shown in Table 2. At day 1 after birth (i.e. 10 days after their mothers were fed standard diet), the fatty acids composition of the liver of pups tissue marginally reflected those of the respective experimental diets, given to their mothers during the first 12 days of pregnancy. The LO diet led to different concentration of fatty acids in pups, i.e. higher ALA and lower ARA concentrations than PO. Conversely, even when the differences in fatty acid profile between newborn vs. adult pups are evident—which probably reflects the different stages of development—the hepatic fatty acid profile of adults (12 months old) were the same, irrespective of the diet provided during the first 12 days of gestation (Table 2).

**Table 1:** Concentrations of specific fatty acids in liver tissue of vigin (V) and pregnant rats (P) at day 12 of fed with diet containing different oils: soybean (SO), olive (OO), fish (FO), linseed (LO) or palm (PO).

	SO		OO		FO		LO		PO	
Fatty acid (FA) mg/g n/group:	Virgin (6)	Pregnant (7)	Virgin (7)	Pregnant (6)	Virgin (7)	Pregnant (6)	Virgin (6)	Pregnant (6)	Virgin (5)	Pregnant (5)
14:0	0.18 ± 0.03^ab^	0.16 ± 0.02^A^	0.19 ± 0.02^ab^	0.25 ± 0.05^AB^	0.29 ± 0.04^b^	0.24 ± 0.04^AB^	0.11± 0.01ª	0.13 ± 0.02^A^	0.17 ± 0.03^ab^	0.32 ± 0.03^B^
16:0	7.72 ± 0.69^a^	5.97 ± 0.36^A^	7.77 ± 0.61^a^	8.65 ± 1.19^A^	8.62 ± 0.61^a^	6.95±0.76^A^	5.01 ± 0.16^b^ *	5.61 ± 0.49^A^	8.59 ± 0.79^a^	11.2 ± 1.18^B^
16:1 (w-7)	0.67 ± 0.11^a^ *	0.46 ± 0.05^A^	0.87 ± 0.10^a^	0.51 ± 0.10^A^	1.41 ± 0.16^a^	0.90±0.13^B^	0.41 ± 0.03^b^	0.41 ± 0.08^A^	0.81 ± 0.19^a^	0.86 ± 0.1^B^
18:0	6.41 ± 0.16^a^	6.19 ± 0.21^AB^	6.22 ± 0.28^a^	6.43 ± 0.57^AB^	5.37 ± 0.21^b^	5.59±0.34^A^	6.09 ± 0.16^a^	6.86 ± 0.2^AB^	6.6 ± 0.2^a^	7.17 ± 0.28^B^
18:1 (w-9)	6.93 ± 0.97^ab^ *	4.35 ± 0.38^A^	15.79 ± 1.66^c^	15.33 ± 3.05^B^	9.11 ± 0.53^b^	6.00±0.64^B^	4.79 ± 0.24^a^	4.7 ± 0.46^A^	9.37 ± 0.99^b^	11.5 ± 1.19^A^
18:2 (w-6)	11.90 ± 1.08^a^	6.81 ± 0.84^A^	4.26 ± 0.51^b^	2.56 ± 0.21^B^	5.95 ± 0.56^b^ *	3.34±0.22^B^	5.54 ± 0.53^b^	4.42 ± 0.45^B^	5.51 ± 0.93^b^	4.2 ± 0.8^B^
18:3 (w-3)	0.69 ± 0.07^a^	0.35 ± 0.05^A^	0.12 ± 0.02^b^	0.06 ± 0.02^A^	0.29 ± 0.02^ab^	0.15±0.02^A^	2.57 ± 0.34^c^	2.38 ± 0.37^B^	0.09 ± 0.02^b^	0.07 ± 0.03^A^
20:4 (w-6)	6.28 ± 0.17^a^	4.93 ± 0.22^A^	6.00 ± 0.30^a^	4.75 ± 0.27^A^	3.08 ± 0.28^b^	2.37±0.20^B^	4.17 ± 0.35^c^	3.04 ± 0.15^C^	6.14 ± 0.41^a^	5.32 ± 0.17^A^
20:5 (w-3)	0.18 ± 0.02^a^	0.11 ± 0.02^A^	0.10 ± 0.01^a^	0.09 ± 0.01^A^	3.12 ± 0.31^b^	2.51±0.34^B^	1.28 ± 0.23^c^	1.75 ± 0.14^C^	0.06 ± 0.01^a^ *	0.15 ± 0.09^A^
22:5 (w-6)	0.11 ± 0.02^a^	0.14 ± 0.01^A^	0.25 ± 0.03^b^	0.30 ± 0.03^B^	0.10 ± 0.01^a^	0.08±0.01^AC^	0.03 ± 0.01^a^	0.04 ± 0.00^C^	0.65 ± 0.08^c^	0.46 ± 0.06^D^
22:5 (w-3)	0.56 ± 0.02^a^	0.39 ± 0.03^A^	0.27 ± 0.01^a^	0.21± 0.01^A^	1.70 ± 0.17^b^	1.47±0.23^B^	0.91± 0.03^c^	0.74 ± 0.05^C^	0.27 ± 0.04^a^	0.27 ± 0.02^A^
22:6 (w-3)	2.11 ± 0.10^a^	1.58 ± 0.08^A^	1.87 ± 0.12^a^	1.42 ± 0.12^A^	7.30 ± 0.52^b^	5.55±0.41^B^	2.02 ± 0.15^a^ *	1.8 ± 0.08^A^	1.62 ± 0.06^a^	1.34 ± 0.09^A^
**SUM OF SATURATED FA**	**14.32 ± 0.86^a^**	**12.33 ± 0.49^A^**	**13.70 ± 0.78^a^**	**15.33 ± 1.75^A^**	**14.28 ± 0.72^a^**	**12.78 ± 1.00^A^**	11.20±0.24^b^*	**12.61 ± 0.62^A^**	**15.37 ± 0.87^a^**	**18.69 ± 1.29^B^**
**SUM OF MONOUNSATURATED FA**	7.69 ± 1.09^ac^*	**4.85 ± 0.42^A^**	**15.68 ± 1.88^b^**	**15.94 ± 3.05^B^**	**11.20 ± 0.67^c^**	**7.49 ± 0.85^A^**	**5.27 ± 0.25^a^**	**5.14 ± 0.51^A^**	**10.27± 1.11^c^**	**12.43 ± 1.24^B^**
**SUM OF POLYUNSATURATED n-6 FA**	**19.22 ± 1.28^a^**	**12.4 6±1.03^A^**	**10.75 ± 0.55^b^**	**8.12 ± 0.50^BC^**	9.57± 0.82^b^**	**6.13 ± 0.37^C^**	**10.18 ± 0.90^b^**	**7.84 ± 0.57^BC^**	**12.99 ± 1.38^b^**	**10.47± 0.95^AB^**
**SUM OF POLYUNSATURATED n-6 FAI w-3**	**3.56 ± 0.15^a^**	**2.45 ±0.12^A^**	**2.30 ± 0.13^a^**	**1.78 ± 0.14^A^**	**12.42 ± 0.92^b^**	**9.69 ± 0.90^B^**	**6.89 ± 0.50^c^**	**6.79 ± 0.41^C^**	**2.07 ± 0.07^a^**	**1.83 ± 0.13^A^**
**SUM OF TOTAL POLYUNSATURATED FA**	**22.79 ±1.37^a^**	**14.91 ±1.10^A^**	**13.06 ± 0.62^b^**	**9.90 ± 0.63^B^**	**21.99 ± 1.45^a^**	**15.82 ± 0.98^A^**	**17.07 ± 0.73^b^**	**14.63 ± 0.96^A^**	**15.06 ± 1.43^a^**	**12.30± 1.01^AB^**

Mean ± SE. SO, Soy oil diet; OO, Olive oil diet; FO, Fish oil diet; LO, Linseed oil diet; PO, Palm oil diet. Lowercase letters at the same row means statistical difference between virgins of all experimental groups. Uppercase letters at the same row means statistical difference between pregnant of all experimental groups with p<0,05. * means statistical difference between virgin and pregnant rats of the same dietary group with:

*: p<0,05;

**: p<0,01.

**Table 2:** Concentrations of specific fatty acids in liver tissue of newborn (1 day old) and male adult pups of rats that fed diets containing different oils during the first 12 days of pregnancy: soybean (SO), olive (OO), fish (FO), linseed (LO) or palm (PO).

	SO		OO		FO		LO		PO	
Fatty acid (FA), mg/g n/group	Pup (6)	Adult-male (12)	Pup (5)	Adult-male (12)	Pup (5)	Adult-male (11)	Pup (5)	Adult-male (10)	Pup (7)	Adult-male (11)
14:0	0.75 ± 0.22^a^ **	0.23 ± 0.03^A^	0.65 ± 0.13ª***	0.21 ± 0.02^A^	0.61 ± 0.07^a^	0.17 ± 0.03^A^	0.45 ± 0.09^a^	0.25 ± 0.05^A^	0.72 ± 0.13^a^ **	0.25 ± 0.03^A^
16:0	21.71 ± 4.70^a^ ***	6.97 ± 0.52^A^	17.42 ± 1.96^a^ *	6.52 ± 0.38^A^	15.75 ± 0.87^a^	6.48 ± 0.42^A^	12.91 ± 0.79^a^	7.27 ± 0.90^A^	15.2 ± 1.31^a^	7.17 ± 0.51^A^
16:1 (w-7)	1.53 ± 0.61^a^	1.41 ± 0.16^A^	1.11 ± 0.23^a^	1.38 ± 0.15^A^	1.08 ± 0.20^a^	0.85 ± 0.17^A^	0.68 ± 0.13^a^	1.52 ± 0.34^A^	1.00 ± 0.07^a^ ***	1.46 ± 0.18^A^
18:0	5.95 ± 0.68^a^ *	3.25 ± 0.15^A^	4.98 ± 0.16^ab^	3.19 ± 0.12^A^	4.88 ± 0.09^ab^	3.33 ± 0.10^A^	4.15 ± 0.30^b^	3.24 ± 0.19^A^	4.59 ± 0.11^b^	3.01 ± 0.09^A^
18:1 (w-9)	11.48 ± 3.05^a^ **	4.92 ± 0.47^A^	10.81 ± 2.46^a^ **	4.63 ± 0.43^A^	9.18 ± 0.77^a^	4.08 ± 0.40^A^	6.70 ± 0.66^a^	5.39 ± 1.01^A^	8.64 ± 1.02^a^	5.03 ± 0.55^A^
18:2 (w-6)	11.72 ± 2.74^a^ **	5.50 ± 0.55^A^	7.61 ± 0.91^a^	5.12 ± 0.32^A^	7.15 ± 0.48^a^	6.11 ± 0.34^A^	6.42 ± 0.43^a^	5.45 ± 0.53^A^	6.58 ± 0.84^a^	5.65 ± 0.49^A^
18:3 (w-3)	0.33 ± 0.11^a^ **	0.12 ± 0.02^A^	0.17 ± 0.04^a^ **	0.10 ± 0.01^A^	0.14 ± 0.01^a^ *	0.13 ± 0.02^A^	0.73 ± 0.15^b^ **	0.13 ± 0.03^A^	0.12 ± 0.02^a^	0.13 ± 0.02^A^
20:4 (w-6)	14.45 ± 1.84^a^ ***	4.91 ± 0.17^A^	10.19 ± 1.20^b^ **	4.69 ± 0.14^A^	7.94 ± 0.60^b^	5.09 ± 0.20^A^	6.16 ± 0.33^b^	4.84 ± 0.26^A^	8.41 ± 0.74 ^b^ ***	4.72 ± 0.11^A^
20:5 (w-3)	0.74 ± 0.23^ab^ ***	0.06 ± 0.01^A^	0.56 ± 0.04^a^ **	0.06 ± 0.00^A^	0.86 ± 0.20^ab^ **	0.06 ± 0.01^A^	1.23 ± 0.23^b^ **	0.07 ± 0.01^A^	0.46 ± 0.04ª**	0.05 ± 0.00^A^
22:5 (w-3)	1.99 ± 0.29^ab^ ***	0.30 ± 0.01^A^	1.72 ± 0.23^a^ **	0.31 ± 0.01^AB^	2.05 ± 0.35^ab^ ***	0.36 ± 0.01^B^	2.80 ± 0.37^b^ ***	0.31 ± 0.02^AB^	1.41 ± 0.09ª**	0.30 ± 0.01^A^
**SUM OF SATURATED FA**	28.41± 5.18^a^***	**10.46±0.66^A^**	23.05±2.18^ab^*	**9.91±0.48^A^**	**21.24±0.10^ab^**	**9.99±0.52^A^**	**17.51±1.09^b^**	**10.75±1.06^A^**	**20.56±1.43^ab^**	**10.42±0.55^A^**
**SUM OF MONOUNSATURATED FA**	13.32 ± 3.69^a^**	**6.34±0.62^A^**	12.30±2.69^a^**	**6.04±0.5^A^**	**10.64±1.01^a^**	**4.96±0.56^A^**	**7.55±0.79^a^**	**6.94±1.3^A^**	**9.93±1.08^a^**	**6.53±0.74^A^**
**SUM OF n-6 POLYUNSATURATED FA**	33.87±6.25^a^***	**11.30±0.73^A^**	23.24±3.22^b^***	**10.60±0.42^A^**	**18.71±0.99^b^**	**12.04±0.47^A^**	**14.86±0.63^b^**	**11.23±0.75^A^**	18.05±1.93^b^***	**11.20±0.55^A^**
**SUM OF n-3 POLYUNSATURATED FA**	8.74±1.14^a^***	**1.47±0.56^A^**	8.36±1.22^a^***	**1.40±0.08^A^**	10.80±1.67^a^***	**1.59±0.03^A^**	10.63±0.95^a^***	**1.57±0.16^A^**	8.36±0.38^a^**	**1.45±0.06^A^**
**SUM OF TOTAL POLYUNSATURATED FA**	42.61±7.27^a^***	**12.77±0.77^A^**	31.61±2.02^b^*	**12.00±0.46^A^**	**29.51±1.40^b^**	**13.63±0.50^A^**	**25.48±1.50^b^**	**12.80±0.86^A^**	27.42±2.03^b^***	**12.66±0.55^A^**

Mean ± SE. SO, Soy oil diet; OO, Olive oil diet; FO, Fish oil diet; LO, Linseed oil diet; PO, Palm oil diet. Lowercase letters at the same row means statistical difference between pups of all experimental groups. Uppercase letters at the same row means statistical difference between adult males with p<0,05. * means statistical difference between pups and adult males of the same experimental group with:

*: p<0,05;

**: p<0,01;

***: p<0,001.

### Liver and adipose tissue miRNA expression in pregnant and virgin rats

**Fig 1:**
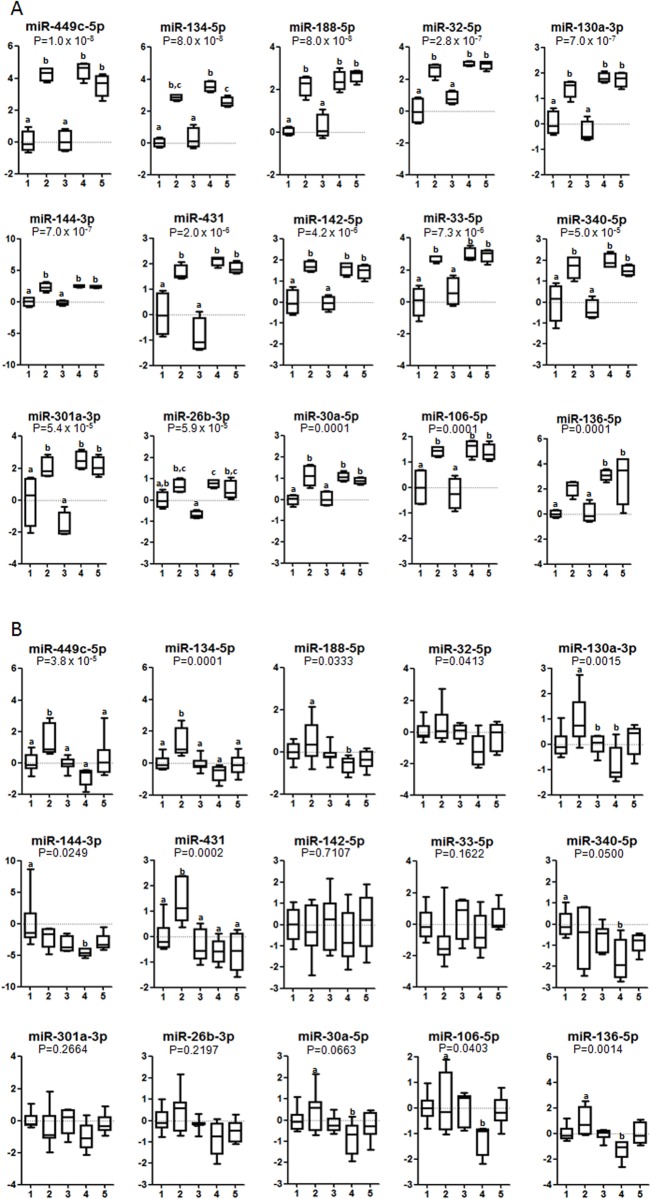
Expression levels of microRNAs in pregnant (A) and virgin rats (B) in liver tissue according to the dietary treatment during 12 days. Data is expressed as fold change (mean ±SD) relative to SO diet group. SO diet, 1; OO diet, 2; FO diet, 3; LO diet, 4; PO diet, 5. Different letters in the same graph means statistical difference (P < 0.05) between dietary groups. Animals per group, in (A) n = 4. In (B): SO (n = 6), OO (n = 7), FO (n = 7), LO (n = 5) and PO (n = 6).

To determine whether dietary consumption of a particular fatty acid during the first 12 days of pregnancy influences the expression of specific miRNAs, we performed an unbiased genome-wide miRNA analysis of liver samples. In pregnant (12 days of diet) liver tissue, 700 miRNAs were measured by qRT-PCR. 15 miRNAs were found to be significantly modulated by any of the source of fatty acids used (Fig. 1A). Namely, pregnant rats fed SO and FO diets during the first 12 days of pregnancy showed significant lower expression of miR-449c-5p, miR-134–5p, miR-188, miR-32, miR130a, miR-144–3p, miR-431, miR-142–5p, miR-33, miR-340–5p, miR-301a, miR-30a, miR-106b, and miR-136–5p, as compared with OO, LO, and PO diets. Similarly, pregnant rats fed SO and FO diets displayed lower miR-26b-3p as compared with OO, LO, and PO diets, although their expression was significantly reduced only for FO compared with OO, LO, and PO diets and for SO compared with LO diet. To test whether the same effect was observed in virgin rats receiving the same type of fatty acids, we evaluated only those 15 miRNAs that changed in their pregnant counterparts. In liver tissues of virgin rats fed the different experimental diets for 12 days, we found that only 10 of the previous miRNA analyzed in pregnant rats remained significantly modulated by any of the dietary fatty acid tested (Fig. 1B). miR-449c-5p, miR-134–5p, miR-130a-3p, and miR-431 expressions was induced after OO compared with other treatments, but at different degrees. In general, miRNA changes in liver of virgin rats were less pronounced than in their pregnant counterparts.

**Fig 2:**
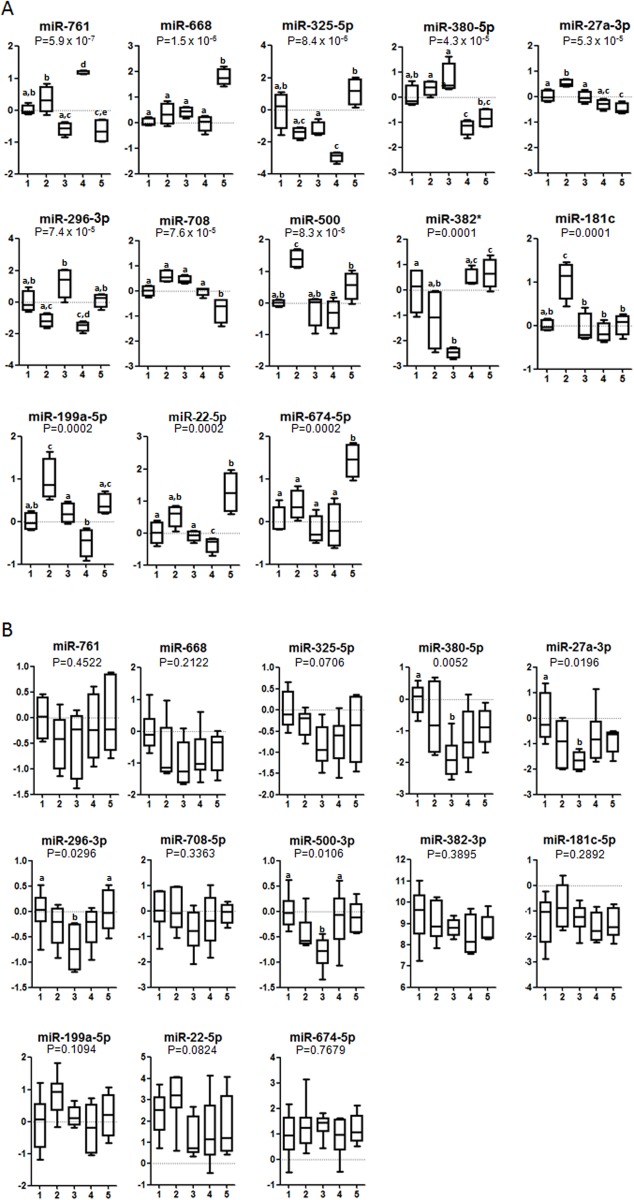
Expression levels of microRNAs in pregnant (A) and virgin (B) rats in lumbar adipose tissue according to the dietary treatment during 12 days. Data is expressed as fold change (mean ±SD) relative to SO diet group. SO diet, 1; OO diet, 2; FO diet, 3; LO diet, 4; PO diet, 5. Different letters in the same graph means statistical difference (P < 0.05) between dietary groups. Animals per group, in (A) n = 4. In (B): SO (n = 6), OO (n = 7), FO (n = 7), LO (n = 6) and PO (n = 5).

To test whether the effect of a particular fatty acid on miRNA expression was observed in other tissues, we also analyzed the adipose tissue whole miRNome by qRT-PCR. In lumbar adipose tissue of pregnant, we observed 13 miRNAs that were significantly modulated by any kind of fatty acids (Fig. 2A). Pregnant rats fed the PO diet during the first 12 days of pregnancy showed significantly higher expression of adipose miR-668 and miR-674–5p and lower expression of miR-708 compared with rats fed the SO, OO, FO, and LO diets. miR-199a-5p and miR-22–5p expression were higher after OO and PA diets compared with LO diet. miR-761 and miR-325 were induced and repressed by LO diet, respectively. miR-500 was induced by OO diet. Again, to test whether the same effects were observed in virgin rats receiving the different diets, we evaluated the 13 miRNAs which significantly changed in pregnant rats. In the adipose tissue of virgin rats, only four of the miRNA previously analyzed in pregnant rats changed significantly (Fig. 2B). After the FO diet, miR-380–5p, miR-27a-3p, and miR-500–3p expressions decreased compared with the SO diet. miR-296–3p expression also decreased after FO compared with the SO and PO diets.

### Liver tissue miRNA expression in newborn (one day-old) and adult pups

**Fig 3:**
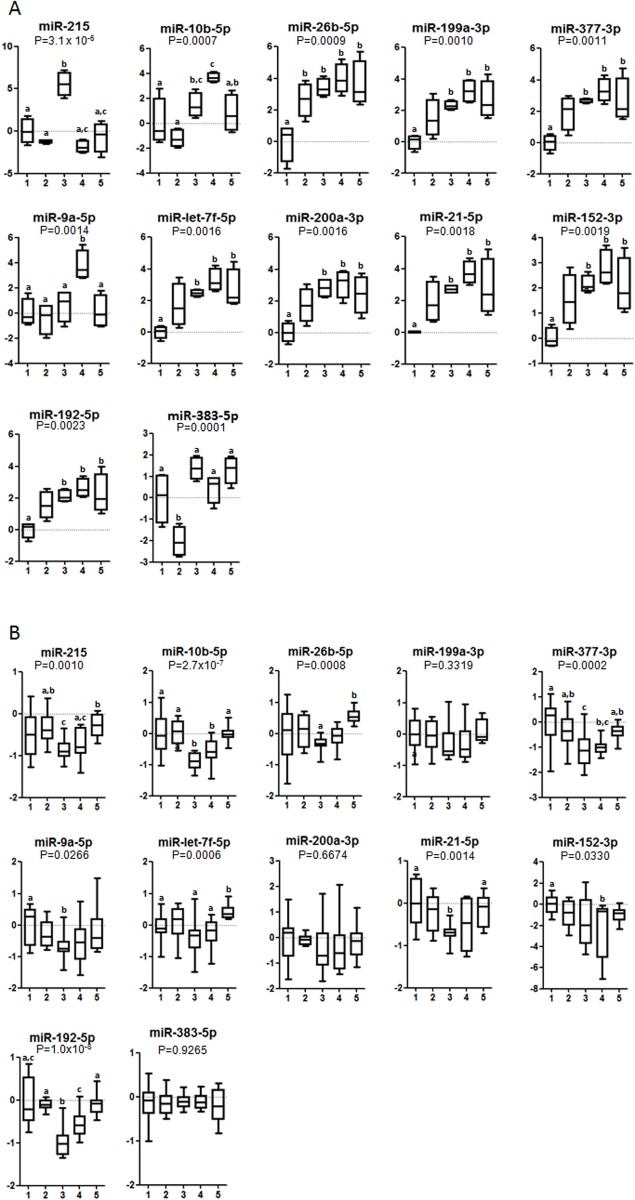
Expression levels of microRNAs in liver of 1 day old (A) and adult pups (B) according to their mother’s dietary treatment during the first 12 days of pregnancy. Data is expressed as fold change (mean ±SD) relative to SO diet group. SO diet, 1; OO diet, 2; FO diet, 3; LO diet, 4; PO diet, 5. Different letters in the same graph means statistical difference (P < 0.05) between dietary groups. Animals per group, in (A) n = 4. In (B): SO (n = 12), OO (n = 12), FO (n = 12), LO (n = 10) and PO (n = 12).

To ascertain whether pups’ miRNAs expression is modulated by different fatty acids consumed by mothers during early pregnancy, we first evaluated the miRNome of the one day-old offspring. Hepatic expression of miRNAs in 1 day old pups is depicted in Fig. 3A. Among the miRNAs measured in the screening, 12 were significantly modified by any of the diets of their mothers. Compared to all other treatments, miR-215 expression was significantly induced by FO consumption, while miR-10b and miR-9a were induced by LO (Fig. 3A). By contrast, miR-26b-5p, miR-199a-3p, miR-377–3p, miR-let-7f-5p, miR-200a-3p, miR-21–5p, miR-152–3p, and miR-192–5p expressions were repressed by SO diet consumption. Moreover, miR-383–5p expression was decreased by the OO diet compared with SO, FO, LO, and PO diets. The 12 miRNAs were validated by real time qRT-PCR in the whole population, and showed similar results (S1 Fig.). We next replicated—in adult pups’liver tissues—the same analyses of the 12 miRNAs that were significantly changed in newborn pup’s liver tissues (Fig. 3B). Adults (12 months old) were chosen as we have previously shown that fish oil group has lower concentration of insulin levels at this age [5]. Moreover, the homeostasis model assessment of insulin resistance and the insulin sensitivity index was generally lower and higher, respectively, for fish oil group than other groups [5]. Adult offspring of rats that were fed the FO diet during the first 12 days of pregnancy showed lower expression of miR-192 compared with SO, OO, LO, and PO diets. Lower expressions of miR-10b-5p and miR-377–3p were also observed in adult pups of rats fed FO diet compared with SO, OO, and PO diets. Similarly, miR-215 expression decreased after FO compared to OO and PO diets and miR-26b-5p expression decreased after FO compared with PO diet and miR-9a-5p after FO compared with SO diet. miR-26b-5p expression was lower after FO compared with PO diet. Finally, miR-let-7f-5p expression was induced after PO compared with SO, FO, and LO diets.

### Liver adult gene expression and miRNA targets

**Fig 4:**
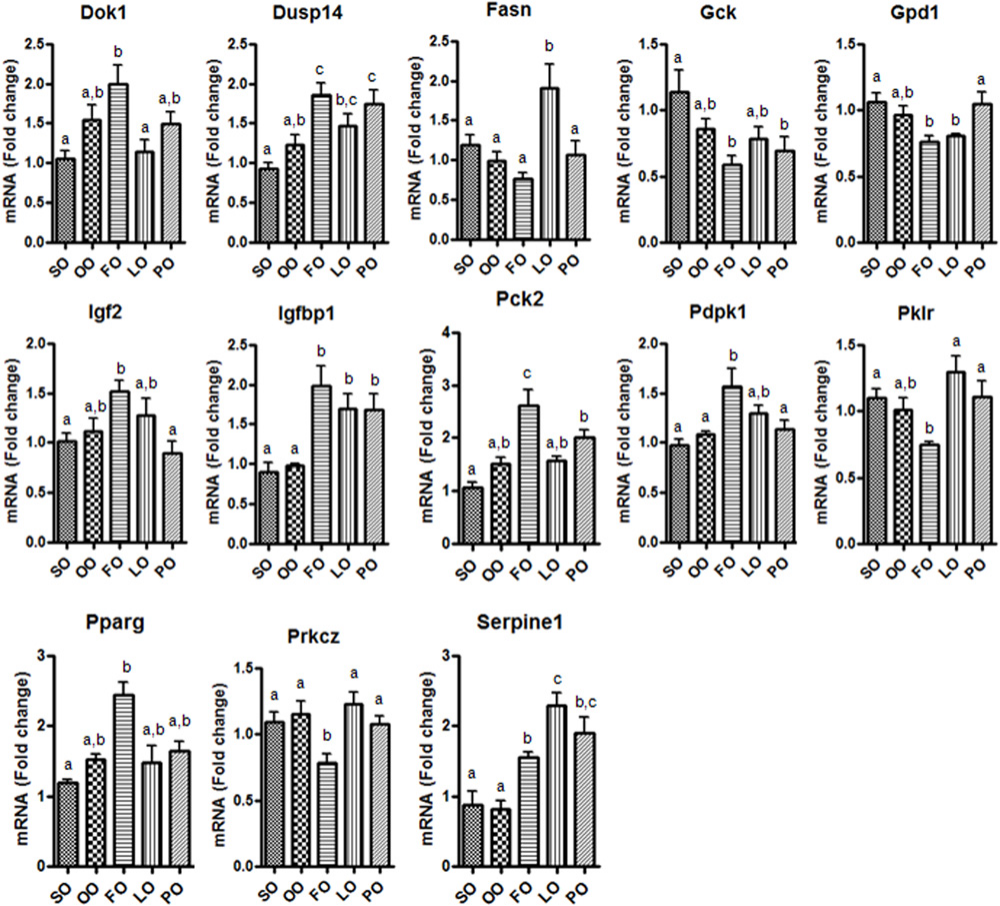
Expression levels selected genes related to insulin signaling in liver of adult pups according to their mother’s dietary treatment during the first 12 days of pregnancy. Data is expressed as fold change (mean ±SEM) relative to soybean (SO) diet group. Olive (OO) diet; fish (FO) diet; linseed (LO) diet; or palm-oil (PO) diet. Different letters in the same graph means statistical difference (P < 0.05) between dietary groups. Animals per group n = 5.

To determine the impact of changes of miRNA expression on their target genes in adult liver samples, we evaluated 88 genes related to insulin signaling. Insulin signaling pathway during adulthood were likely to be influenced by early nutrition during pregnancy [5], but mechanism for these effects were not evaluated. Some of the genes within this panel were predicted to be target by some of the miRNAs differentially regulated by the different fatty acids, as analyzed by miRWalk database [29]. As expected, only a small amount of genes were differentially modulated by the diet received during early pregnancy (Fig. 4 and S2A and S2B Fig.), some of which were modulated preferentially by fish oil diet. Reduced expression of miR-192, miR-215 and miR-10b in liver samples of rat from fish oil group (Fig. 3B) resulted in a de-repression of their targets plasminogen activator inhibitor type 1 (Serpine1) and insulin-like grow factor 2 (Igf2), as both genes have predicted binding sites for these three miRNAs. Indicating that miRNAs contribute to their regulation. However, we do not discard that other modulated genes (Fig. 4) might be also regulated by other miRNAs not evaluated in these samples, or regulated by other miRNA-independent mechanisms.

## Discussion

Understanding the molecular consequences of specific fatty acid supplementations during the first half of pregnancy—in terms of offspring development—is important to promote and/or reinforce recommendations of the consumption of adequate amounts of a particular type of fatty acid. This study provides in vivo evidence that different miRNAs are induced or repressed in the liver and adipose tissues of pregnant and virgin rats according to diets added with different types of fatty acids. We firstly demonstrated that miRNA expression in the liver is distinctively regulated by the type of fatty acid consumed and, secondly, we found that miRNAs expression in adipose tissue is different from their expression in liver, after the intake of diets with different fatty acid profiles. These results suggest that it is important to assess the expression of miRNAs tissue-by-tissue, in order to assess the final effect of the intake of diets with different types of fatty acids. Not surprisingly, tissue distribution or development stage strongly influences miRNA function. Indeed, some miRNAs are highly expressed in or restricted to a specific tissue or cell type [30,31]. As an example, liver and adipose tissue miRNA analyses performed in pregnant rats by matching their virgin counterparts did not show similar significant changes in their expression after consumption of the same diets with different fatty acid profile. These data clearly indicate that pregnancy plays a discernible role in miRNA expression regulation. It is important to note that the primary role of miRNAs is to “fine-tune” gene expression, but their function becomes particularly important under conditions of stress [15]. In this respect, pregnancy could be considered as a physiological “stress” state, where different biochemical and hormonal changes take place to ensure the survival of the fetus.

We also report that the type of fatty acid consumed by mothers during the first 12 days of pregnancy influence miRNAs expression in the liver of offspring at birth and even at 12 months of age. Of note, we did not detect miRNAs that were mutually and significantly modulated by the same type of dietary fat in livers of pregnant, virgin rats, newborn and adult pups. Previous studies observed that maternal high fat diets during pregnancy and lactation modulates hepatic miRNA expression in offspring [7,8]. However, those studies did not analyze the expression of miRNAs in pregnant rats. In our study, the fact that normolipidic diets with different fatty acids were consumed only during the first 12 days of pregnancy and not during the full pregnancy and lactation period or adult life could partially explain the differences observed in the expression levels of miRNAs among pregnant rats and offspring. Also, maternal consumption of a high-fat diet during lactation, but not during pregnancy, causes obesity and glucose intolerance in adult offspring and developmental alterations involving hypothalamic proopiomelanocortin (POMC) neurons, a key hypothalamic neuronal projections governing systemic metabolism [4]. Indeed, targeted deletion of insulin receptors from POMC cells partially restored their projections to the paraventricular nucleus while also reversing the associated obesity phenotype [4]. However, it is important to note that our study tried to approximate the most physiological conditions observed in healthy pregnancy. Therefore, we did not provide high-fat diets, but, rather, we administered a normal rodent diet (chow) in which the habitual quantity of fat (20% of total energy intake) was the solely dietary component that was differentially changed. Finally, we only fed rats during the first 12 days of pregnancy, when a large proportion of dietary fatty acids are stored in adipose tissue for later utilization in fetal development [27].

Our results might bear physio-pathological relevance. As an example, the hepatic expression of miRNAs in the adult offspring may partially explain the delay of the decline in insulin sensitivity observed in male offspring after the intake of high amounts of n-3 fatty acids during early pregnancy [5]. In particular, Sardinha *et al*. [5] observed that plasma insulin concentrations of male adult offspring (12 months old) fed a FO diet during the first 12 days of pregnancy were lower than those of adults fed OO or PO diets. Moreover, the AUC for insulin in response to an oral glucose load at 8 months of age was lower in the FO group than in the OO, LO, or PO groups; that of SO were lower than in both OO and PO groups. Likewise, we observed a decrease in the expression of several hepatic miRNAs, namely miR-192–5p, miR-10b-5p, miR-377–3p, and miR-215 after FO compared with OO and PO diets and miR-21–5p and mir-26b-5p after FO compared with PO diets. These data could partially explain the plasma insulin concentrations results mentioned above [5], because most of these miRNAs and their targets are related to insulin and glucose metabolism.

From an epigenetic viewpoint, the kind of fatty acid consumed by the mother during early pregnancy could affect gene regulation by inducing (or repressing) transcription of specific genes during critical ontogenic periods, during which tissue-specific marks may be initiated. Even though our work is in accordance with previous studies indicating that environmental exposure, i.e. diet in parents influence gene expression in offspring [12,13,32], the precise molecular mechanism(s) by which these miRNAs are regulated by a specific type of fatty acids remains to be fully elucidated.

Among the validated gene targets of some of these miRNAs, there is the insulin grown factor pathway (Igf-1r and Igf-1), which is targeted by miR-192 and miR-215 [33]. These miRNAs were found to be induced in diabetes [34]. Moreover, Igf2 and Serpine1 are among predicted targets of this family of miR-192/215. miR-377 was also found to be upregulated in diabetic nephropathy [35]. miR-21 was found to be induced in response to high glucose [36,37] and targets the tumor suppressor protein phosphatase and tensin homolog deleted in chromosome 10 (Pten), which plays important roles in liver glucose metabolism, lipogenesis, steatohepatitis, and hepatocellular carcinoma [38–40]. Other validated target of miR-21 are the fas ligand (Faslg) [41], Pdcd4 [42], and Tiam1 [43] which are also related to diabetes [42,44]. miR-26b is required for adipogenesis and target Pten and Adam17 [45,46]; moreover, it is also regulated by glucose levels [47]. In synthesis, some validated target of these miRNAs suggest that—by regulating different genes—fatty acid-induced miRNA changes may in part explain the observed effects on hepatic insulin-signaling pathways in the offspring [5]. However, whether the changes on these miRNAs in the liver of rats receiving FO during early pregnancy are the only responsible for the observed effects is yet to be fully clarified (S3 Fig.). Indeed, a feature of miRNA function is that a single miRNA can simultaneously bind and repress multiple target genes; likewise, the possibility that a single mRNA be targeted by multiple miRNAs provides mechanisms to synchronize the coordinated regulation of a large suite of transcripts that govern an entire biological process, in turn resulting in strong phenotypic output [15].

Pharmacological modulation of miRNA activity has received special attention from the scientific and clinical audience as a novel approach to treat disease by modulating entire biological pathways. Indeed, numerous pharmacological tools have been developed to target miRNA pathways [48–50]; promising results have been reported in human clinical trials to either inhibit their action [51] (Santaris Pharma A/S) or as miRNA replacement therapy (Clinicaltrial.gov; NCT01829971, Mirna Therapeutics, Inc). Dietary modulation of miRNA action is an interesting alternative to the former approaches [22,52,53]. Our results show for the first time that different types of fatty acid during early pregnancy not only modulate the expression of miRNAs in liver and adipose tissue of pregnant rats but also influence short- and long-term miRNA expression in their offspring.

In conclusion, our data add novel in vivo evidence to the notion that fatty acids can modulate miRNA expression [23,52] in a tissue-specific and temporally-restrained manner. We also show that the type of fatty acid consumed by the mother during early pregnancy elicits epigenetic mechanisms through miRNAs modulation in offspring [7,32]. One important feature of our contribution is that we comparatively assessed the effects of five diets containing different fatty acid profiles. The precise molecular mechanism(s) underlying the changes in miRNA expression in pregnant mothers and their adult offspring induced by a particular type of fatty acid deserve further investigation. Yet, our data suggest that dietary fatty acid modulation of miRNA expression might theoretically be a viable option to accompany current pharmacological therapy targeting endogenous miRNAs.

## Supporting Information

S1 Table Composition of experimental diets. SO, Soy oil diet; OO, Olive oil diet; FO, Fish oil diet; LO, Linseed oil diet; PO, Palm oil diet; ND, not detected.
(DOCX)

S1 Fig Validation of expression levels of microRNAs in liver of 1 day old according to their mother’s dietary treatment during the first 12 days of pregnancy. Individual assays were performed by real time qRT-PCR. Data is expressed as fold change (mean ±SD) relative to SO diet group. SO diet, 1; OO diet, 2; FO diet, 3; LO diet, 4; PO diet, 5. Different letters in the same graph means statistical difference (P < 0.05) between dietary groups. Animals per group: SO (n = 5), OO (n = 4), FO (n = 4), LO (n = 4) and PO (n = 6).
(TIF)

S2 Fig (A–B) Expression levels selected genes related to insulin signaling in liver of adult pups according to their mother’s dietary treatment during the first 12 days of pregnancy. Data is expressed as fold change (mean ±SEM) relative to soybean (SO) diet group. Olive (OO) diet; fish (FO) diet; linseed (LO) diet; or palm-oil (PO) diet. Animals per group n = 5.
(TIF)

S3 Fig Maternal fish oil diet during early gestation alters hepatic expression of key miRNAs related to insulin signaling in the adult offspring. A maternal FO diet during 12 days gestation might regulate different genes related to insulin signaling and their predicted and validated targets in the liver tissue of adult offspring. Igf2 and Serpine1 are targets of miR-192/215 and miR-10b family. miR-21 targets Pten, which is involved in liver glucose metabolism and other related process. Other validated targets of miR-21 related to diabetes are the fas ligand (Faslg), Pdcd4 and Tiam1. miR-377 is also related to diabetic nephropathy [35]. miR-26b, which is regulated by glucose levels [47], is required for adipogenesis and target Pten and Adam17 [45,46].
(TIF)
