# Supplementary material for: Consumption of Distinct Dietary Lipids during Early Pregnancy Differentially Modulates the Expression of microRNAs in Mothers and Offspring
Source: PLoS One. 2015 Feb 11;10(2):e0117858. doi: 10.1371/journal.pone.0117858 (PMC4324823; doi:10.1371/journal.pone.0117858)
Supplement: S1 Table — SO, Soy oil diet; OO, Olive oil diet; FO, Fish oil diet; LO, Linseed oil diet; PO, Palm oil diet; ND, not detected. (DOCX) [file pone.0117858.s001.docx]

**Table S1**. Composition of experimental diets

|  | **SO** | **OO** | **FO** | **LO** | **PO** |
| --- | --- | --- | --- | --- | --- |
| **Constituents g/kg of diet** |  |  |  |  |  |
| Casein (vitamin free) | 200 | 200 | 200 | 200 | 200 |
| Cornstarch | 397.4 | 397.4 | 397.4 | 397.4 | 397.4 |
| Dextrinized cornstarch | 132.0 | 132.0 | 132.0 | 132.0 | 132.0 |
| Sucrose | 80.0 | 80.0 | 80.0 | 80.0 | 80.0 |
| Cellulose | 50.0 | 50.0 | 50.0 | 50.0 | 50.0 |
| Salt mix^a^ | 10.0 | 10.0 | 10.0 | 10.0 | 10.0 |
| Vitamin mix^b^ | 35.0 | 35.0 | 35.0 | 35.0 | 35.0 |
| Β-Choline | 2.5 | 2.5 | 2.5 | 2.5 | 2.5 |
| Butylhydroquinone-BHT | 0.01 | 0.01 | 0.01 | 0.01 | 0.01 |
| Soy oil | 90.0 | - | - | - | 10.0 |
| Olive oil | - | 90.0 | - | - | - |
| Sunflower oil | - | - | 10.0 | 10.0 | - |
| Fish oil | - | - | 80.0 | - | - |
| Linseed oil | - | - | - | 80.0 | - |
| Palm oil | - | - | - | - | 80.0 |
| **Fatty acids mg/g of diet**^c^ |  |  |  |  |  |
| Myristic (14:0) | 0.39 | 0.33 | 3.84 | 0.38 | 0.95 |
| Palmitic (16:0) | 8.23 | 8.29 | 8.06 | 5.71 | 26.85 |
| Palmitoleic (16:1, n-7) | 0.23 | 0.61 | 6.61 | 0.16 | 0.46 |
| Stearic (18:0) | 3.46 | 2.69 | 1.89 | 3.90 | 3.62 |
| Oleic (18:1, n-9) | 15.46 | 48.34 | 16.17 | 15.99 | 24.41 |
| Linoleic (18:2, n-6) | 31.49 | 4.15 | 6.28 | 13.76 | 10.42 |
| Arachidonic (20:4, n-6) | 0.02 | ND | 0.36 | 0.03 | ND |
| α-linolenic (18:3, n-3) | 4.06 | 0.45 | 0.86 | 31.21 | 0.92 |
| Eicosapentaenoic (20:5, n-3) | 0.06 | 0.11 | 7.65 | 0.21 | 0.08 |
| Docosahexaenoic (22:6, n-3) | ND | ND | 7.72 | 0.08 | ND |

SO, Soy oil diet; OO, Olive oil diet; FO, Fish oil diet; LO, Linseed oil diet; PO, Palm oil diet; ND, not detected.

^a^ Salt mix (g/kg diet): Copper sulfate 0.1; ammonium molybdate 0.026; sodium iodate 0.0003; potassium chromate 0.028; zinc sulfate 0.091; calcium hydrogen phosphate 0.145; ammonium ferrous sulfate 2.338; magnesium sulfate 3.37; manganese sulfate 1.125; sodium chloride 4; calcium carbonate 9.89; potassium dihydrogen phosphate 14.75.

^b^ Vitamin mix (mg/kg diet): retinyl palmitate 2.4; cholecalciferol 0.025; menadione sodium bisulfite 0.8; biotin 0.22; cyanocobalamin 0.01; riboflavin 6.6; thiamin hydrochloride 6.6; tocopherol acetate 100.

^c^ Values correspond to the mean of three separate samples processed independently.
