# Supplementary figures and images for: Consumption of Distinct Dietary Lipids during Early Pregnancy Differentially Modulates the Expression of microRNAs in Mothers and Offspring
Source: PLoS One. 2015 Feb 11;10(2):e0117858. doi: 10.1371/journal.pone.0117858 (PMC4324823; doi:10.1371/journal.pone.0117858)

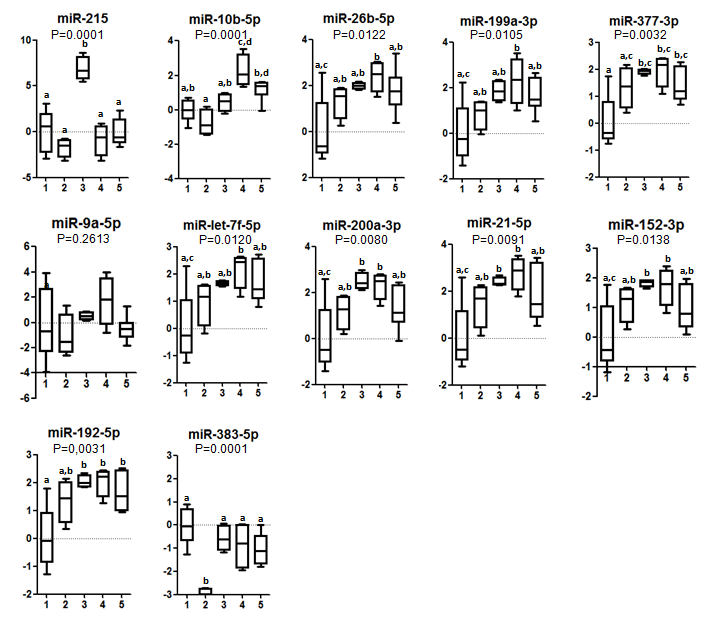

Supplement: S1 Fig — Individual assays were performed by real time qRT-PCR. Data is expressed as fold change (mean ±SD) relative to SO diet group. SO diet, 1; OO diet, 2; FO diet, 3; LO diet, 4; PO diet, 5. Different letters in the same graph means statistical difference (P < 0.05) between dietary groups. Animals per group: SO (n = 5), OO (n = 4), FO (n = 4), LO (n = 4) and PO (n = 6). (TIF) [file pone.0117858.s002.tif]

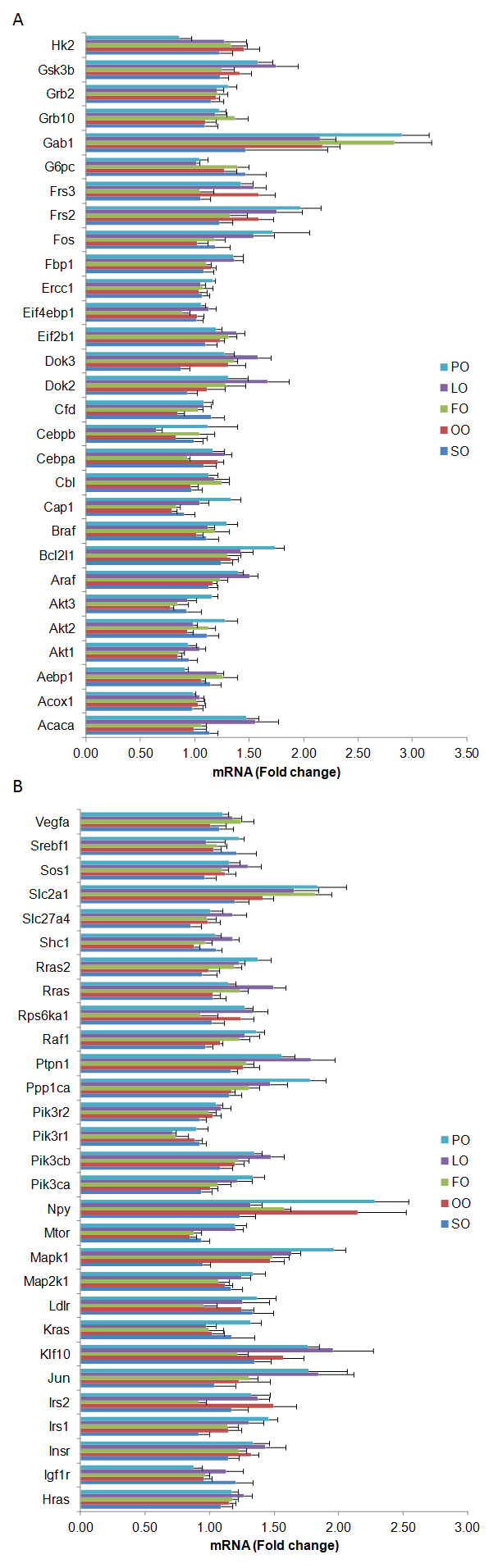

Supplement: S2 Fig — Data is expressed as fold change (mean ±SEM) relative to soybean (SO) diet group. Olive (OO) diet; fish (FO) diet; linseed (LO) diet; or palm-oil (PO) diet. Animals per group n = 5. (TIF) [file pone.0117858.s003.tif]

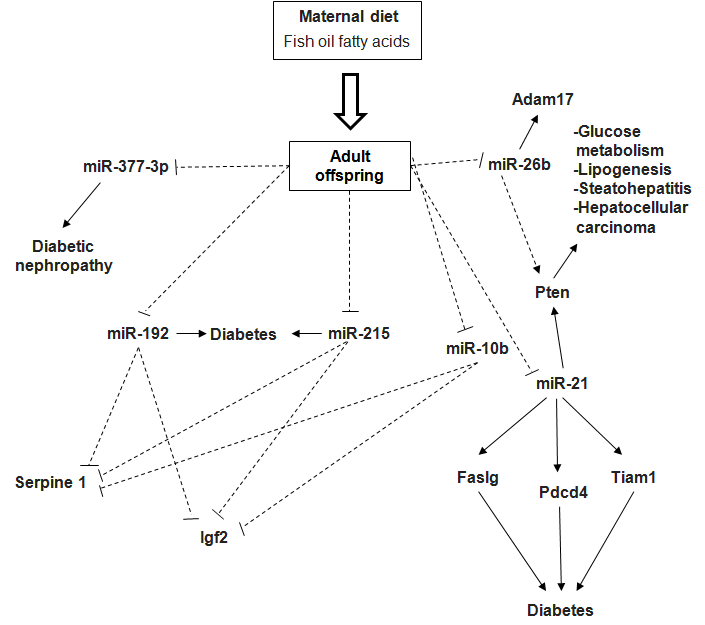

Supplement: S3 Fig — A maternal FO diet during 12 days gestation might regulate different genes related to insulin signaling and their predicted and validated targets in the liver tissue of adult offspring. Igf2 and Serpine1 are targets of miR-192/215 and miR-10b family. miR-21 targets Pten, which is involved in liver glucose metabolism and other related process. Other validated targets of miR-21 related to diabetes are the fas ligand (Faslg), Pdcd4 and Tiam1. miR-377 is also related to diabetic nephropathy [35]. miR-26b, which is regulated by glucose levels [47], is required for adipogenesis and target Pten and Adam17 [45,46]. (TIF) [file pone.0117858.s004.tif]
